# A strategy for evaluating the impact of processing of Chinese meteria medica on meridian tropism: the influence of salt-water processing of phellodendri chinensis cortex on renal transport proteins

**DOI:** 10.3389/fphar.2025.1558298

**Published:** 2025-04-07

**Authors:** Yang Chen, Fan Zhang, Wenjing Ren, Yue Zhou, Shiru Jiang, Shuo Zhang, Gui Xu, Xiutong Ge, Hui Gao

**Affiliations:** School of Pharmacy, Liaoning University of Traditional Chinese Medicine, Dalian, Liaoning, China

**Keywords:** phellodendri chinensis cortex, salt-water processing, drug-protein interactions, renal targeting efficacy, transporter proteins

## Abstract

**Introduction:**

This study elucidated the potential mechanisms by which Phellodendri Chinensis Cortex with salt-water processing (SPC) enhances renal targeting efficacy, through investigating the effects of Phellodendri Chinensis Cortex (PC) on renal uptake and efflux transport capabilities before and after salt-water processing.

**Methods:**

This study employed molecular docking, UPLC-TDQ-MS/MS, BCA, Western Blotting, and RT-PCR to assess the effects of raw Phellodendri Chinensis Cortex (RPC), SPC, berberine (BBR), and berberrubine (BBRR) on the transport capacity and expression of renal transport proteins OAT1, OAT3, OCT2, MATE1, MATE2K, P-gp, and MRP2 in HEK-293 cells.

**Results:**

Analyses demonstrated that BBR and BBRR exhibited a strong affinity for OCT2, P-gp, MRP2. Compared to RPC, SPC can increase the uptake capacity and expression of OCT2, while it can decrease efflux capacity and expression of P-gp and MRP2. Simultaneously, BBRR showed similar effects on OCT2, P-gp, and MRP2, compared to BBR. Therefore, the enhanced renal targeting effect of SPC can be attributed to the differential impact of the partial conversion of BBR to BBRR on the transport capacity of the renal transporters OCT2, P-gp, and MRP2.

**Conclusion:**

This study investigated the interactions between renal transporter proteins and drugs, with the objective of elucidating the mechanism by which SPC enhances renal targeting efficacy. The findings of this study offer new insights and methodologies for exploring the effects of Processing of Chinese Materia Medica (PCMM) on the meridian tropism of other traditional Chinese medicines (TCMs).

## 1 Introduction

According to the traditional Chinese medicine theory, traditional Chinese medicines (TCMs) need to be processed into herbal decoction pieces through Processing of Chinese Materia Medica (PCMM) before clinical application. The unique technology and methods of PCMM enable TCMs to reduce toxicity and enhance efficiency, thereby altering medicinal properties and influencing meridian tropism. Meridian tropism refers to the phenomenon in which a drug selectively exerts therapeutic effects on particular regions of the human body. It suggests that the drug’s action is mainly associated with specific viscera or meridians, while showing minimum or no effect on other viscera and meridians. For instance, Bupleuri Radix [*Apiaceae*; *Bupleurum chinense* DC.], when processed with vinegar, is effective for syndromes associated with liver meridian disorders characterized by distending pain in the chest and hypochondrium, as well as irregular menstruation related to the liver meridian (Zhao and Zhao, 2022). Moreover, Anemarrhenae Rhizoma [*Asparagaceae*; *Anemarrhena asphodeloides* Bunge], when processed with salt-water, is indicated for syndromes associated with kidney-meridian disorders characterized by tidal fever, night sweats, cough, and hemoptysis (Fan et al., 2020). The traditional Chinese medicine theory, combined with PCMM, evidently influences meridian tropism, as demonstrated by the above-mentioned examples. However, the theory remains in a traditional expression phase, lacking support from modern scientific and technological methods and experimental data.

**FIGURE 1:**
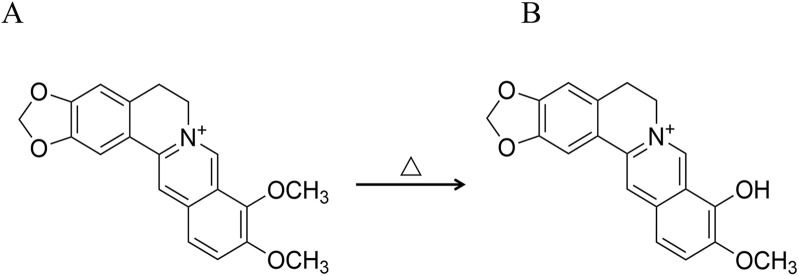
Reaction formula for the conversion of BBR to BBRR. **(A)** BBR, **(B)** BBRR.

In the theory of traditional Chinese medicine, Phellodendri Chinensis cortex (PC) is known to be bitter and cold, acting on the kidney and bladder, with the ability to clear heat and dry dampness, purge fire, relieve steaming, and remove toxins (Jia et al., 2013). In the clinical practice of TCMs, PC can be processed with various methods, including honey-processing, salt-water processing, and wine-processing, each of which produces distinct therapeutic properties (Cheng et al., 2019). The theory of traditional Chinese medicine posits that the salt-water processing of PC markedly improves its effectiveness in nourishing the kidneys, eliminating ministerial fire, and alleviating asthenic fever. Consequently, PC with salt-water processing (SPC) is extensively utilized in Chinese clinical settings for treating conditions stemming from the kidney-yin deficiency pattern (KYDP), such as tidal fever and night sweating, dysentery, and tinnitus spermatorrhea (China Pharmacopoeia Commission, 2020). From the perspective of traditional Chinese medicine, the increased efficacy of SPC is ascribed to the salt-water processing of the drug, which improves renal targeting efficacy. This perspective originated during the era of the *Ming Dynasty* (Liu P. P. et al., 2013), as documented in texts like *Enlightenment on Materia Medica*. These texts highlight the use of salt-water to direct medicines to the kidney meridian tropism, thereby enhancing the softening and dispersing of knots. In addition, *Pharmaceutical Discrimination* also mentions the use of stir-fried drugs with salt-water to primarily reduce yin-fire and manage kidney-water levels. The aforementioned contents can be summarized as the traditional PCMM theory of “entering into the kidney by processing with salt-water”. PC is known for its diverse active metabolites, in addition, PC with salt-water processing induces structural changes in certain metabolites, leading to the transformation of some active metabolites into others (Que et al., 2022). A notable example is the partial conversion of the predominant alkaloid berberine (BBR) into berberrubine (BBRR) (Qi et al., 2010; Zhang et al., 2013; Li et al., 2021) (Figure 1). The BBR (Cat. No.: A0152, purity ≥ 98%) and BBRR (Cat. No.: M1107AS, purity ≥ 98%) were purchased from Manster Biotechnology Co., Ltd. (Chengdu China) in this study.

Studies have identified some functional transmembrane transporter proteins in the human kidney (Zhang and Liu, 2010), which exhibit differences in binding substrates. The specific recognition of substrates by these proteins is crucial for renal secretion and reabsorption of drugs, which influence the level of drug absorption and metabolism in the body. These proteins can be categorized into uptake and efflux transporters based on their contribution to intracellular drug accumulation (Morrissey et al., 2012). The former, including Organic Cation Transporter 2 (OCT2), Organic Anion Transporter 1 (OAT1), and Organic Anion Transporter 3 (OAT3), are pivotal for transporting drugs from the extracellular environment into the cell, thereby increasing intracellular drug accumulation. The latter, including P-glycoprotein (P-gp), Multidrug-resistance-associated protein 2 (MRP2), and Multidrug and Toxin Extrusion Proteins (MATEs), are responsible for drug extrusion from the cell, decreasing intracellular drug accumulation. Consequently, by examining the effects of raw Phellodendri Chinensis Cortex (RPC) and SPC on renal transporter proteins with distinct transport directions and specific substrate recognition functions, the renal uptake and efflux of drugs can be simulated. These examinations provide a basis for evaluating the impact of SPC on the renal targeting capability of drugs.

This study employs molecular docking techniques to predict the conformational relationship and affinities of BBR and BBRR with OAT1, OAT3, OCT2, P-gp, MATE1, MATE2K, and MRP2 in the kidney. It also evaluates the effects of RPC, SPC, BBR, and BBRR on the concentration of various substrates in cell lysates or extracellular medium from HEK-293 cells using UPLC-TDQ-MS/MS, and predicts the transport capacity of renal proteins using the BCA method. This study also assesses the effects of RPC, SPC, BBR, and BBRR on the expression levels of transporter proteins and their mRNAs under the same conditions in HEK-293 cells using Western blotting and RT-PCR. Furthermore, this study investigates the effects of RPC, SPC, and the transformation of their single active metabolites on the function of renal transporter proteins and also analyzes the mechanism through which PC with salt-water processing enhances renal targeting efficacy from the perspective of renal transporter proteins. Finally, it further explains the PCMM theory of “entering into the kidney by processing with salt-water.”

## 2 Materials and methods

### 2.1 Plant materials

Phellodendri Chinensis Cortex [*Rutaceae*; *Phellodendron chinense* C.K. Schneid.], known as Huangbo in Chinese, was purchased as PC herbal decoction pieces from Ju Yao Tang Pharmaceutical Co., Ltd. (Cat. No: 2307003, Anguo, China) and identified by Professor Zhai Yanjun from Liaoning University of Traditional Chinese Medicine based on their morphological characteristics and physicochemical properties, according to the guidelines outlined in *Chinese Pharmacopoeia of 2020 edition*. Voucher specimens of PC have been deposited in the herbarium of Chinese Materia Medica processing engineering center of Liaoning province, Liaoning University of Traditional Chinese Medicine.

### 2.2 Drugs and reagents

Estrone-3-sulfate (Cat. No.: N161B232492, purity ≥ 98%), vinblastine (Cat. No.: X29O11Y128962, purity ≥ 98%) were purchased from Yuanye Biotechnology Co., Ltd. (shanghai, China), Trypsin (Cat. No.: MA0232), pre-stained iridescent protein Maker (Cat. No.: MA0354) were purchased from Meilun Biotechnology (Dalian China). DMEM high glucose medium (Cat. No.: 8123207), PBS buffer salt solution (Cat. No.: 20210927) were purchased from Gibco (Waltham, Massachusetts, United States), fetal bovine serum (Cat. No.: ST200913) was purchased from PAN (Aden Bach, Free State of Bavaria, Germany), penicillin-streptomycin-Amphotericin B Mixed Triple Antibody Solution (Cat. No.: 20220519JH), 20×TBST buffer (Cat. No.: T1082), Whole Protein Lysis Kit (Cat. No.: BC3710), TRIzol Lysis Solution (Cat. No.: 15596018CN), ECL PLUS Ultra-Sensitive Luminescent Solution (Cat. No.: PE0010), dimethyl sulfoxide (Cat. No.: 710N0310), metformin (Cat. No.: D9351, purity≥98%), methotrexate (Cat. No.: M8971, purity≥98%), rhodamine123 (Cat. No.: R8030, purity≥98%), topotecan (Cat. No.: IT1030, purity≥98%) were purchased from Solarbio Science and Technology Co. Ltd (Beijing, China), SDS-PAGE Protein Sampling Buffer 5× (Cat. No.: P0015L) was purchased from Beyotime Bio-Tech Ltd (Shanghai, China), OCT2 rabbit anti-polyclonal antibody (Cat. No.: 10867-2-AP), OAT1 rabbit anti-polyclonal antibody (Cat. No.: 26574-1-AP), OAT3 rabbit anti-polyclonal antibody (Cat. No.: 16844-1-AP), P-gp rabbit anti-polyclonal antibody (Cat. No.: 22336-1-AP), MATE1 rabbit anti-polyclonal antibody (Cat. No.: 20,898), P-gp rabbit anti-polyclonal antibody (Cat. No.: 22336-1-AP), MATE1 rabbit anti-polyclonal antibody (Cat. No.: 20898-1-AP), MATE2K rabbit anti-polyclonal antibody (Cat. No.: 26873-1-AP), MRP2 rabbit anti-polyclonal antibody (Cat. No.: 29261-1-AP), Goat Anti-Rabbit IgG II Antibody (Cat. No.: 20001097) were purchased from the Proteintech (Wuhan, China). Human GAPDH Gene Primer Pair (Cat. No.: AG11723), SYBR green kit (Cat. No.: AG11718), reverse transcription premix kit (Cat. No.: AG11705) were purchased from Acres Bioengineering Co. Ltd. (Changsha, China), mass spectrometry-grade methanol, chromatography-grade methanol, and acetonitrile were purchased from Sigma (Billerica, Massachusetts, German).

### 2.3 Instruments

Waters ACQUITY UPLC/Xevo TQD tandem mass spectrometer for ultra-high performance liquid chromatography (Waters Corporation, Milford, Massachusetts, United States), Mettler Toledo AE240-type analytical balance with a precision of one hundred thousandths (Mettler-Toledo Corporation, Greifensee, Switzerland), ZHJH-C1112B-type biological safety cabinet (Zhi Cheng, Shanghai, China), SHA-C-type constant-temperature oscillator (Changzhou Guohua, Changzhou, China), MCO-15AC-type CO_2_ incubator (Sanyo, Osaka, Japan), LX-800-type microcentrifuge (Sangong Biological, Shanghai, China), laboratory centrifuge (Sigma, Billerica, Massachusetts, United States), NIB-100-type inverted optical microscope (Yongxin, Ningbo, China), MK3-type enzyme-linked immunosorbent assay reader (Thermo Fisher Scientific, Waltham, Massachusetts, United States), Milli-Q ultrapure water system (Millipore, Billerica, Massachusetts, United States), KQ-250E-type medical ultrasonic cleaner (Ultrasonic Corporation, Kunshan, China), SL-62508-type horizontal shaker (Qilimbeier Corporation, Haimen, China), L96G-type PCR machine (Longji Corporation, Hangzhou, China), electrophoresis apparatus and gel tank (BIO-RAD Corporation, Hercules, California, United States), and G:BOX Gel Imager (Syngene, Bangalore, India).

### 2.4 Molecular docking

The chemical structures of BBR (PubChem CID: 2353) and BBRR (PubChem CID: 72704) were downloaded from the PubChem (https://pubchem.ncbi.nlm.nih.gov/) and the protein structures of OAT1 (Uniprot ID: Q4U2R8), OAT3 (Q8TCC7), OCT2 (Uniprot ID: O15244), MATE1 (Uniprot ID: Q96FL8), MATE2K (Uniprot ID: Q86VL8), P-gp (Uniprot ID: Q6RVA0), and MRP2 (Uniprot ID: Q92887) were downloaded from the Alphafold (https://alphafold.ebi.ac.uk/). The chemical structures of BBR and BBRR were set as ligands by using Autodock Tools (https://autodock.scripps.edu/) after adding hydrogenation and setting torsion bonds, and the above protein’s structures were set as receptors after adding hydrogenation and dewater. After preparations, molecular docking operations were conducted, and visualization processing was performed using PyMOL (https://pymol.org/). Finally, the binding energy from the docking was used to compare the affinity of RPC and SPC with the above-mentioned proteins.

### 2.5 Preparation of RPC and SPC

RPC preparation: An appropriate amount of PC was collected, purified, softened, and then cut into shreds. The shredded PC was subsequently dried and collected.

SPC preparation: The shredded PC was purified, mixed with 2% salt-water (2 g of salt and 30 mL of water for every 100 g of PC), moistened for 2 h, heated in a pot at 150°C–160°C, stir-baked for 6 min, cooled, and collected (Zhang et al., 2022).

All the different processed products of PC used in this study were prepared according to the guidelines outlined in *Chinese Pharmacopoeia of 2020 edition*, ensuring their compliance with established regulatory requirements.

### 2.6 Preparation of drug-containing serum and various solutions

Drug-containing serum preparation: According to previous studies, the serum preparations have been reported (Bu et al., 2024; Chen et al., 2024; Yang et al., 2024). In brief, thirty male SD rats of SPF grade were randomly assigned to three groups: a blank group (10 rats), an RPC-containing plasma group (10 rats), and an SPC-containing plasma group (10 rats). In the RPC and SPC groups, the prepared gavage solution of RPC and SPC (0.96 g/mL) were given to rats at a dose of 1 mL/100 g (Ren et al., 2024). For the blank group, the rats were given the same dose of distilled water. On the seventh consecutive day, blood was collected from the abdominal aorta 2 h after gavage and allowed to clot at room temperature for 1 h. The blood was then centrifuged at 3,500 rpm for 15 min to separate the serum, which was subsequently inactivated for 30 min in a water bath at 56°C. The serum was filtered through a 0.22-μm micropore membrane to remove bacteria, yielding the normal rat serum and the RPC or SPC-containing serum. These serums were then frozen and stored at −20°C for further analysis.

BBR and BBRR solutions preparation: An appropriate amount of BBR and BBRR was weighed and dissolved in medium with 10% normal rat serum to prepare the administration solutions at a concentration of 10 μM, according to previous studies (Zhu et al., 2011; Yang et al., 2016).

Substrate solutions preparation: An appropriate amount of metformin (MET) (Xiao et al., 2018), methotrexate (MTX) (Huo et al., 2020), estrone-3-sulfate (E3S) (Jia et al., 2016), rhodamine 123 (Rho123) (Pohl et al., 2002), topotecan (TOP) (Wittwer et al., 2013), and vinblastine (VBL) (Van Aubel et al., 1999) were appropriately weighed and dissolved in a few dimethyl sulfoxide (DMSO). The solutions were then thoroughly mixed with transport buffer (containing 118 mM NaCl, 23.8 mM NaHCO_3_, 4.8 mM KCl, 1.0 mM KH_2_PO_4_, 1.2 mM MgSO_4_, 12.5 mM HEPES, 5.0 mM glucose, and 1.5 mM CaCl_2_, pH 7.4) (Nies et al., 2017) to prepare the administration solutions at a concentration of 10 μM.

Reference substance solutions preparation: Metformin, methotrexate, estrone-3-sulfate, rhodamine123, topotecan, and vinblastine were precisely weighed. Methanol was mixed with each reference substance to prepare a series of reference substance solutions at a uniform concentration of 10 μg/mL for subsequent analytical assays. This ensured the precision and consistency of the experimental results.

### 2.7 Cell culture

HEK-293 cells (purchased from Priscilla Life Science and Technology, Wuhan, China) were cultured in DMEM high glucose complete medium, supplemented with 10% FBS, 1% penicillin-streptomycin-amphotericin B solution, and 5% CO_2_, at 37°C under constant temperature and humidity. Cells were harvested for subsequent assays after reaching 80%–90% confluence, with the culture medium refreshed every day.

### 2.8 Cell uptake assays

For this experiment, HEK-293 cells were seeded into 12-well plates and incubated with 5% CO_2_ at 37°C for 24 h. For the blank control, 1 mL of medium with 10% normal rat serum was added, while experimental groups received 1 mL of medium with 10% serum containing RPC or SPC, and 1 mL of medium with BBR solutions or BBRR solutions. After continuing the incubation for 0, 15, 30, 45, and 60 min, 10 μM of metformin, methotrexate, and estrone-3-sulfate were added to the experimental groups, respectively, and incubated again for 30 min, with three parallel wells in each group. When the incubation was completed, the cells were washed three times using PBS, and the cell samples from each group and the third PBS washing were collected. After adding 1 mL of pure water to the samples, they were freeze-thaw three times at −80°C and room temperature. After centrifugation at 12,000 rpm for 20 min at low temperature, the supernatant was collected, and 1 mL of methanol was added for protein precipitation, followed by a second centrifugation at 12,000 rpm for 20 min. The supernatant obtained post-centrifugation, along with the third wash solution of PBS, was collected and evaporated to dryness using a nitrogen stream. Subsequently, 500 μL of methanol was added to reconstitute the residue, followed by vortexing for 2 min. Finally, the resulting supernatant was filtered by a 0.22-μm micropore membrane and stored at 4°C for UPLC-TDQ-MS/MS analysis.

### 2.9 Cell efflux assay

For this experiment, HEK-293 cells were seeded into 12-well plates and incubated with 5% CO_2_ at 37°C for 24 h. After the medium was aspirated and discarded, 10 μM of rhodamine 123, topotecan, and vinblastine were added to each well, followed by a 30-min incubation. Subsequently, 1 mL of medium with 10% normal rat serum was added to each well of the blank control group, while 1 mL of medium with 10% serum containing RPC or SPC, and BBR solutions or BBRR solutions were added to each well of the experimental groups. The cells were further incubated for 0, 15, 30, 45, and 60 min, with three replicate wells for each time point. The extracellular medium and the third PBS washing from both the blank control and experimental groups were then collected and evaporated to dryness under a nitrogen stream. Finally, 500 μL of methanol was added to reconstitute the residue, followed by vortexing for 2 min. The resulting supernatant was then filtered by a 0.22-μm micropore membrane and stored at 4°C for UPLC-TDQ-MS/MS analysis. Table 1 shows the grouping of the experiments.

**TABLE 1:** Grouping cells uptake and efflux substrate experiments for each transporter protein in HEK-293 cells.

Transporter protein type	Transporter protein in the kidney	RPC + specific probe substrate	SPC + specific probe substrate	BBR + specific probe substrate	BBRR + specific probe substrate
Uptake transporter proteins	OCT2	RPC + MET	SPC + MET	BBR + MET	BBRR + MET
	OAT1	RPC + MTX	SPC + MTX	BBR + MTX	BBRR + MTX
	OAT3	RPC + E3S	SPC + E3S	BBR + E3S	BBRR + E3S
Efflux transporter proteins	P-gp	RPC + Rho123	SPC + Rho123	BBR + Rho123	BBRR + Rho123
	MATE1/2K	RPC + TOP	SPC + TOP	BBR + TOP	BBRR + TOP
	MRP2	RPC + VBL	SPC + VBL	BBR + VBL	BBRR + VBL

### 2.10 Protein transport assay

The uptake of metformin, methotrexate, and estrone-3-sulfate and the efflux of rhodamine, topotecan, and vinblastine in cell lysates or extracellular media was quantified with UPLC-TDQ-MS/MS. The uptake proteins or efflux proteins were also normalized using a BCA protein assay kit (Solarbio, Beijing, China). Then the transport ability of OAT1, OAT3, OCT2, MATE1, MATE2K, P-gp, and MRP2 was predicted by comparing the differences in substrate accumulation content (the amount of each transporter protein transporting the corresponding substrate per unit mass) in each dosing group under the same conditions.

### 2.11 UPLC-TDQ-MS/MS analysis

A Waters ACQUITY UPLC system equipped with a Xevo TQ-S mass spectrometer was employed to identify substrates for cellular uptake and efflux. The liquid chromatography separations were using an ACQUITY UPLC BEH C_18_ column (2.1 mm × 100 mm, 1.7 μm). The mobile phase consisted of 0.1% formic acid in water (A) and 0.1% formic acid in acetonitrile (B). The flow rate was maintained at 0.2 mL/min, the column temperature was set to 35°C, and the injection volume was 2 μL. The gradient elution for determining methotrexate was set at 0–12 min, 56% A, 44% B, and for determining metformin, estrone-3-sulfate, rhodamine 123, topotecan, and vinblastine were set at 0 min, 90% A, 10% B; 6 min, 0% A, 100% B; 6–12 min, 0% A, 100% B.

**FIGURE 2:**
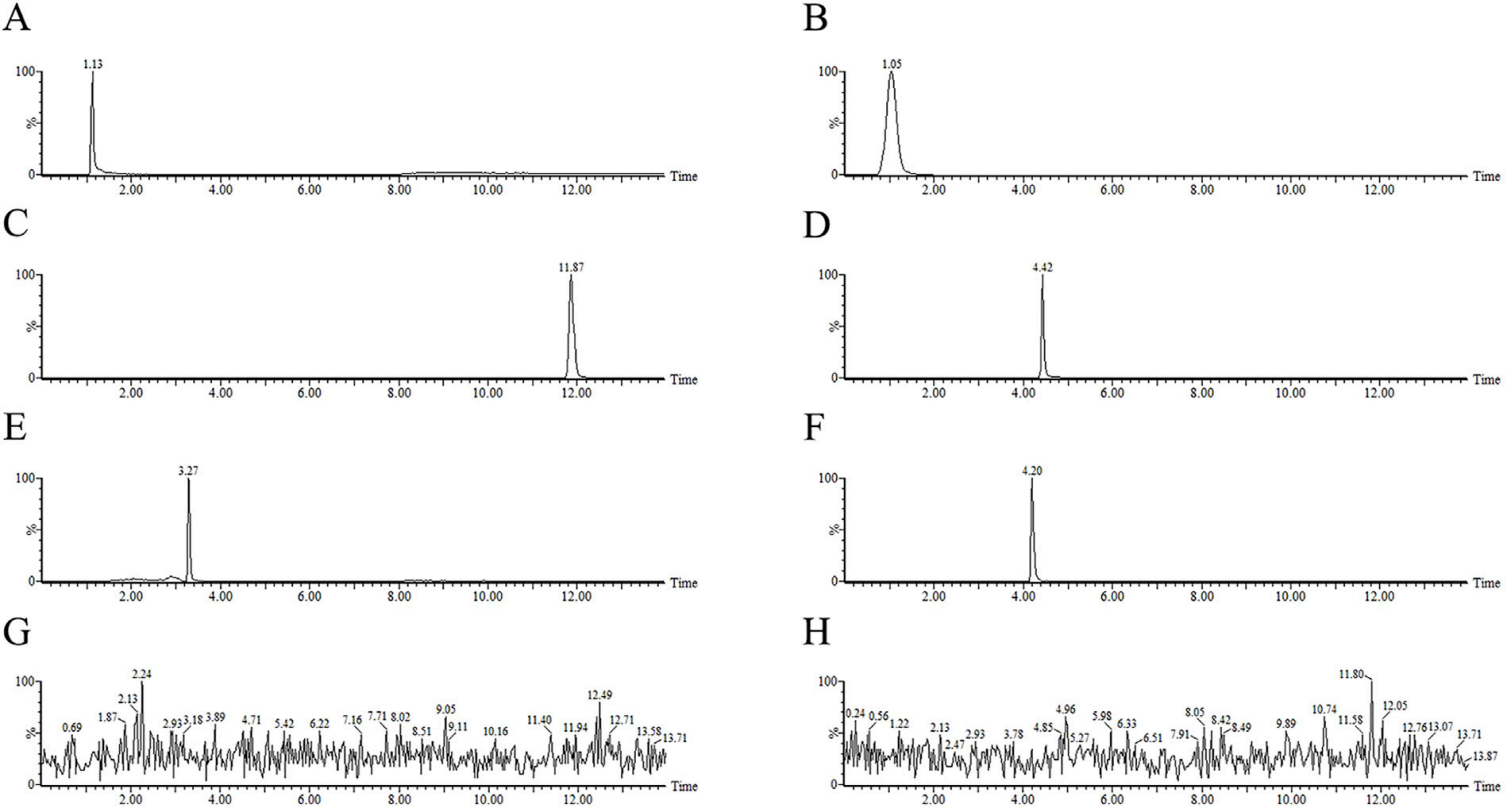
The total ion chromatogram (TIC) of tested samples of metformin, methotrexate, estrone-3-sulfate, rhodamine 123, topotecan, vinblastine in MRM mode from HEK-293 cells uptake and efflux assay. **(A)** metformin **(B)** methotrexate **(C)** estrone-3-sulfate **(D)** rhodamine 123 **(E)** topotecan **(F)** vinblastine standards **(G)** third PBS washing **(H)** blank control.

This analysis employed the electrospray ionization (ESI) source to acquire spectra in positive ion scan mode, covering a m/z range of 50–1000. The capillary voltage was optimized at 3.0 kV with the source temperature and the desolvation temperature set at 150°C and 450°C, respectively. The desolvation gas flow was maintained at 900 L/h and the cone gas flow was 50 L/h. The multi-reaction monitoring (MRM) mode was used to simultaneously scan multiple ions and detect the targets. Table 2 presents the detailed mass spectrometric parameters for the metformin, methotrexate, estrone-3-sulfate, rhodamine 123, topotecan, and vinblastine, and Figure 2 shows the ion chromatograms of these metabolites in HEK-293 cell lysates and extracellular medium samples from each experimental group after administration, as well as the total ion chromatograms of the uptake and efflux of experimental, blank control groups and the third PBS washing.

**TABLE 2:** Mass spectrometric parameters for metformin, methotrexate, estrone-3-sulfate, rhodamine 123, topotecan, and vinblastine in MRM mode.

Specific probe substrates	Parention m/z	Daughter ion m/z	Cone voltage/v	Collision energy e/v
MET	130.1	60.1	18	14
MTX	455.2	308.2	34	20
E3S	351.1	271.3	29	28
Rho123	345.1	285.1	50	40
TOP	422.2	377.2	38	17
VBL	811.4	224.2	50	40

### 2.12 Validation of UPLC-TDQ-MS/MS conditions for quantification

A comparison of the chromatograms between the groups revealed no significant interferences of endogenous or exogenous in MRM mode, which validates this analysis. Aliquots from each reference substance solution, prepared at a concentration of 10 μg/mL, were aspirated in equal volumes and sequentially diluted by a factor of 10 using methanol in a stepwise manner. Quantification for both the HEK-293 cell uptake and efflux assays was conducted using the external standard method via standard curves. Table 3 shows coefficients of determination (*R* ^2^) for the substrate standards exceeded 0.99, signifying excellent linearity. The quantification limit of the analysis was determined to be 1.024 × 10^−3^ ng/mL, with linearity established across the range of 1 × 10^−3^ to 1 × 10^4^ ng/mL. After the samples were reconstituted according to “2.8” and “2.9”, the relative standard deviation (RSD) values of the inter-day precision of each substrate standard ranged from 1.76% to 7.64% with an accuracy ranging from 94.08% to 103.14%. Intra-day precision RSD values were within the range of 2.11%–6.33% with an accuracy of 95.12% and 104.73%. Moreover, the extraction recoveries for the method oscillated between 96.53% and 104.65%. After storage at 4°C for 24 h, the reproducibility and stability of each substrate standard and samples of uptake or efflux experiments were satisfactory, suggesting no significant interference from other factors.

**TABLE 3:** Linear relationships of reference substance solutions.

Substrates	Linear regression curve	*R* ^2^
MET	y = 2.01479x+450.504	0.9991
MTX	y = 25.4069x + 259.939	0.9919
E3S	y = 114.644x + 47,371	0.9983
Rho123	y = 67.5675x + 8009.48	0.9955
TOP	y = 0.887177x −0.994332	0.9977
VBL	y = 0.400917x + 5.23785	0.9991

### 2.13 Western blotting assay

Western blotting assay was used to further investigate the effects of PC before and after salt-water processing on the expression of transporters OAT1, OAT3, OCT2, P-gp, MATE1, MATE2K, and MRP2 in the kidney. HEK-293 cells were seeded into a 6-well plate at the density of 2 × 10^5^ cells/mL and then incubated with 5% CO_2_ at 37°C for 24 h. The cells were categorized into a blank control group, which was supplemented with medium containing 10% normal rat serum, and experimental groups, which were administrated with medium containing 10% RPC or SPC serum, and BBR or BBRR solutions. After incubating drug solutions for 24 h, the cells were harvested and lysed in RIPA buffer for 20 min on ice. The supernatant resulting from centrifugation (12,000 rmp, 10 min) was normalized for protein concentration using the BCA protein assay kit. Then SDS protein loading buffer (5×) was added to the protein samples in a 4:1 ratio. After mixing thoroughly, the mixtures were denatured by boiling them at 99°C for 15 min. A 16 μL aliquot of protein samples was electrophoresed on 10% or 6% SDS-PAGE gels (80 V for 30 min, followed by 120 V for 60 min), and transferred onto 0.45 µm PVDF membrane at 400 mA for 30 min. The membrane was then blocked with 5% BSA for 1 hour at room temperature and incubated overnight with the primary antibody at 4°C. After being incubated with the secondary antibody for 1 h at room temperature, the membrane was washed three times with 1× TBST. Finally, the membrane was incubated with an ECL luminescent solution for 3–10 min, and the data were analyzed in ImageJ.

### 2.14 RT-PCR assay

In this assay, HEK-293 cells were seeded into a 6-well plate at the density of 2 × 10^5^ cells/mL and then incubated with 5% CO_2_ and 37°C for 24 h. The cells were categorized into a blank control group, which was supplemented with medium containing 10% normal rat serum, and experimental groups, which received medium containing 10% RPC or SPC serum, and BBR or BBRR solutions. After the RPC, SPC, BBR, and BBRR solutions were incubated for 24 h, the cells were harvested and lysed in a TRIzol reagent. Total RNA was extracted from the HEK-293 cells using an RNAex kit. Reverse transcription was performed using a reverse transcription kit with a total reaction volume of 10 μL, employing primer ratios as specified in the experimental design (cDNA: forward primer: reverse primer: reverse transcription kit = 3: 1: 1: 5). The PCR amplification protocol included 40 cycles of initial denaturation at 95°C for 10 min, annealing at 60°C for 1 min, and extension at 60°C for 1 min. Table 4 shows the primer sequences for OCT2, OAT1, OAT3, P-gp, MATE1, MATE2K, and MRP2.

**TABLE 4:** Primer sequences of OCT2, OAT1, OAT3, P-gp, MATE1, MATE2K, MRP2 genes.

Gene name	Direction	Primer sequence	Primer length (bp)
OCT2	Forward	5′-GAC​CAT​CGA​GGA​AGC​CGA​AA-3′	20
	Reverse	5′-GCA​GCA​ACG​GTC​TCT​CTT​CT-3′	20
OAT1	Forward	5′-GGA​GCC​AAA​TTG​AGT​ATG​GAG​GTA-3′	24
	Reverse	5′-ATA​GTA​TGC​AAA​GCT​AGT​GGC​AA-3′	23
OAT3	Forward	5′-TAC​ACC​TTT​GGC​CAG​TTC​ATT​CT-3′	23
	Reverse	5′-CTT​CGA​GGA​CTT​TCC​AGA​CAA​GA-3′	23
P-gp	Forward	5′-GGG​ATG​GTC​AGT​GTT​GAT​GGA-3′	21
	Reverse	5′-GCT​ATC​GTG​GTG​GCA​AAC​AAT​A-3′	22
MATE1	Forward	5′-TCA​ACC​AGG​GAA​TTG​TAC​TGC-3′	21
	Reverse	5′-CAG​AGC​CTA​TCA​CCC​CAA​GA-3′	20
MATE2K	Forward	5′-TCT​TCG​GCA​TGT​GAC​ACC​TTG-3′	21
	Reverse	5′-CCT​GGG​TCA​ACC​TGG​ACA​C-3′	19
MRP2	Forward	5′-CCC​TGC​TGT​TCG​ATA​TAC​CAA​TC-3′	23
	Reverse	5′-TCG​AGA​GAA​TCC​AGA​ATA​GGG​AC-3′	23

### 2.15 Statistical analysis

Statistical processing and analysis were performed in SPSS 17.0. Data were presented as mean ± standard deviation (x̅±SD) and analyzed using one-way analysis of variance (ANOVA) with the least significant difference (LSD). The MassLynx 4.1 workstation was utilized to analyze the trends in substrate contents within HEK-293 cells, while graphical representations were crafted using GraphPad Prism 10. *p* < 0.05 was set to indicate statistical significance, and *p* < 0.01 was considered to reflect a highly significant difference.

## 3 Results

### 3.1 Molecular docking visualization

**FIGURE 3:**
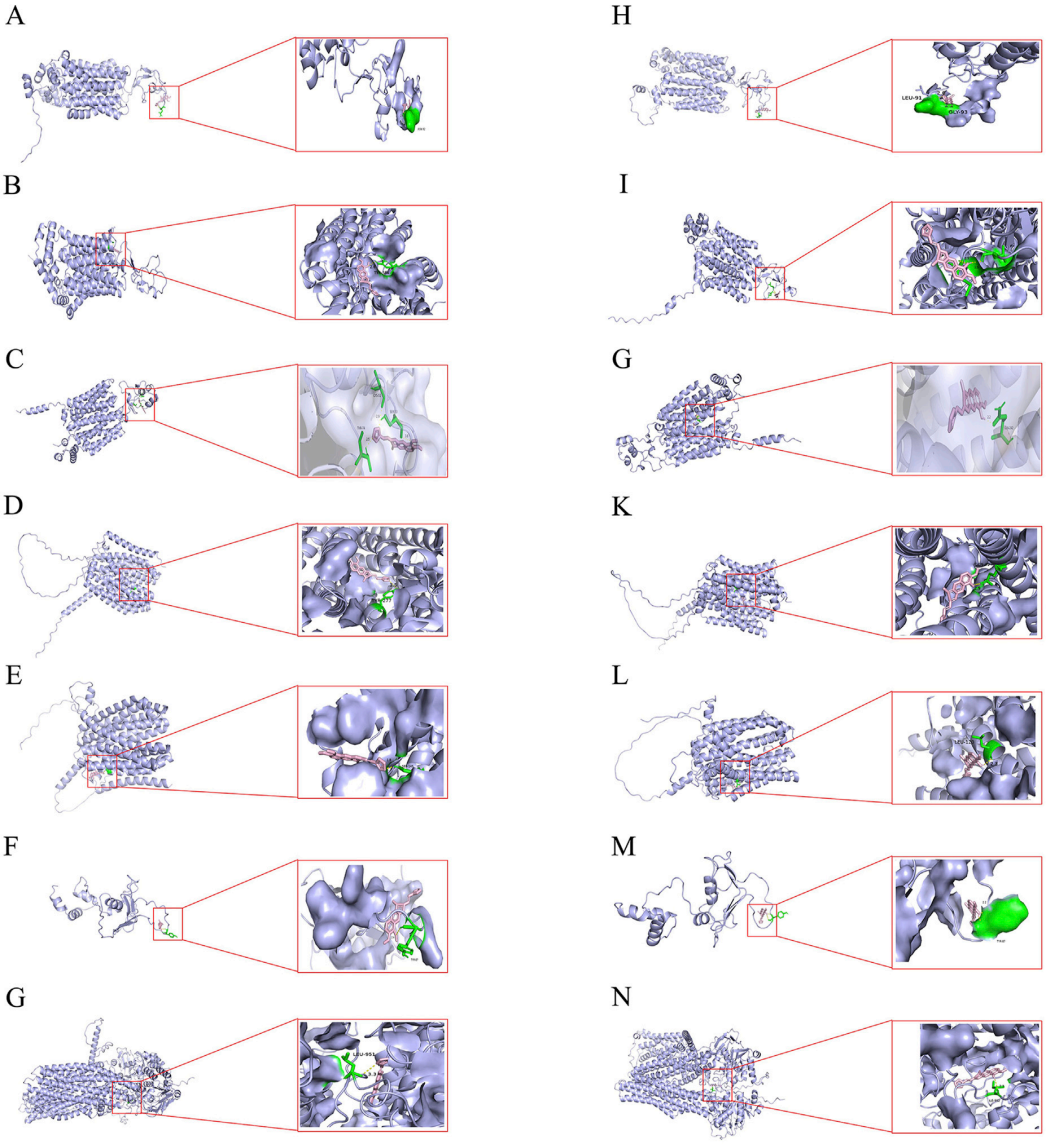
Molecular docking illustrations of BBR and BBRR with seven transport proteins respectively. **(A)** BBR docked with OAT1. **(B)** BBR docked with OAT3. **(C)** BBR docked with OCT2. **(D)** BBR docked with MATE1. **(E)** BBR docked with MATE2K. **(F)** BBR docked with P‐gp. **(G)** BBR docked with MRP2. **(H)** BBRR docked with OAT1. **(I)** BBRR docked with OAT3. **(J)** BBRR docked with OCT2. **(K)** BBRR docked with MATE1. **(L)** BBRR docked with MATE2K. **(M)** BBRR docked with P‐gp. **(N)** BBRR docked with MRP2.

Molecular docking visualization showed the substantial binding affinity of BBR and BBRR for the organic cation transporter OCT2 and the efflux proteins MATE1, P-gp, and MRP2 (Table 5). The specific binding sites and the interaction patterns of BBR and BBRR with these seven transporter proteins are graphically depicted in Figure 3.

**TABLE 5:** Values of the binding energies of berberine and berberrubine to seven transporter proteins in the kidney (Kcal/mol).

Target proteins	Metabolites	
	Berberine	Berberrubine
OAT1	−1.81	−5.81
OAT3	−1.81	−5.74
OCT2	−6.22	−7.62
MATE1	−8.77	−8.56
MATE2K	−6.23	−5.81
P-gp	−7.83	−7.47
MRP2	−8.11	−7.88

### 3.2 Effect of cell uptake and efflux assay

**FIGURE 4:**
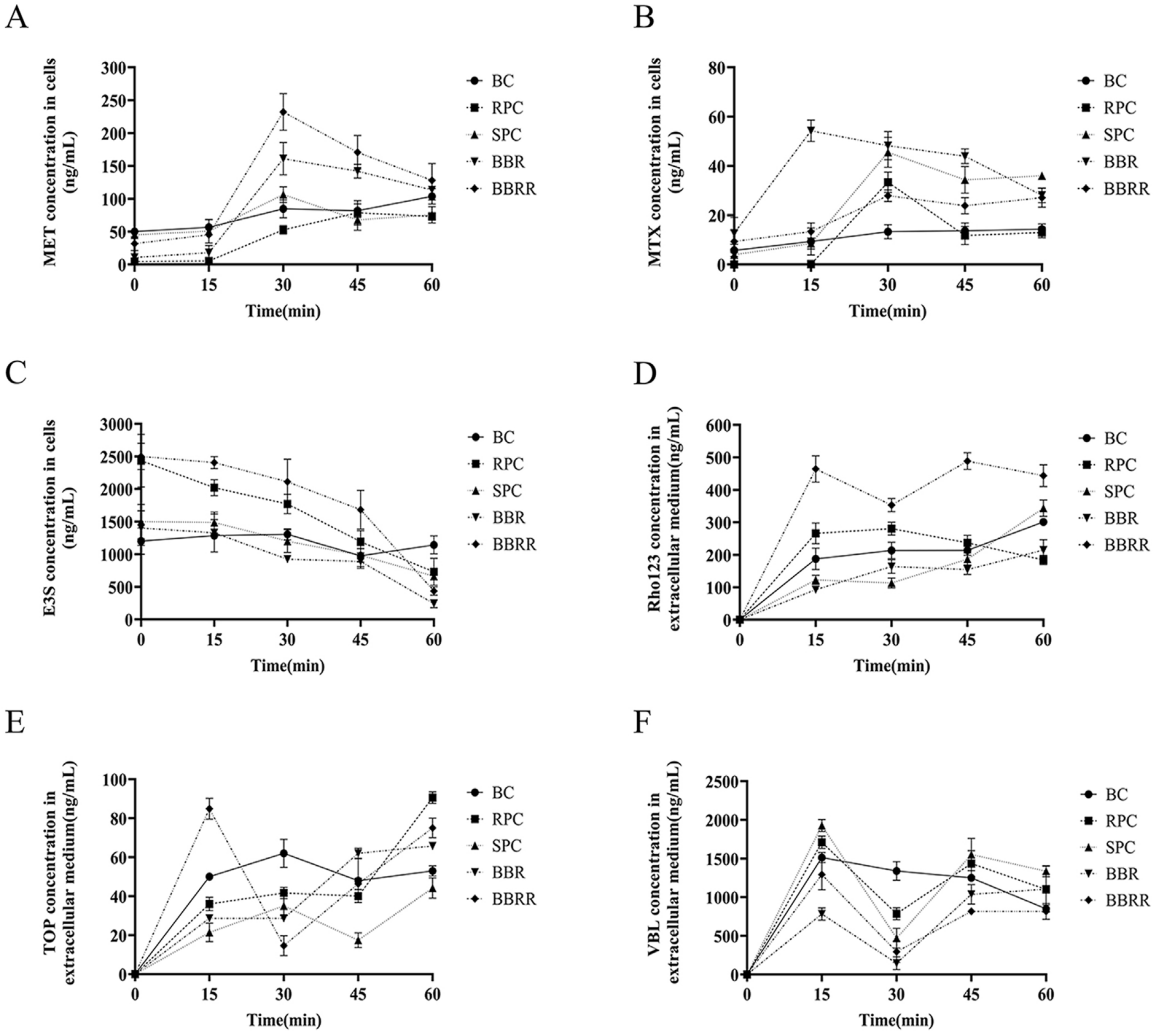
Trends of metformin, methotrexate, estrone-3-sulfate, topotecan, rhodamine 123, and vinblastine concentration under different dosing time conditions in cells lysates or extracellular medium from HEK-293 cells. Blank control (BC), raw Phellodendri Chinensis cortex (RPC), Phellodendri Chinensis Cortex with salt-water processing (SPC), berberine (BBR), berberrubine (BBRR). **(A)** The trend of MET concentration changes mediated by OCT2. **(B)** The trend of MTX concentration changes mediated by OAT1. **(C)** The trend of E3S concentration changes mediated by OAT3. **(D)** The trend of Rho123 concentration changes mediated by P-gp. **(E)** The trend of TOP concentration changes mediated by MATE1 and MATE2K. **(F)** The trend of VBL concentration changes mediated by MRP2 (n = 3).

The analysis of intracellular substrate concentration in cell lysates or extracellular media from HEK-293 cells treated with RPC, SPC, BBR, and BBRR revealed distinct trends. This result also revealed that PC affected the renal uptake and efflux of transporter proteins before and after salt-water processing. Figure 4 shows the trend of concentration of every substrate in HEK-293 cells. There was a substantial increase in the intracellular concentration of these substrates for metformin in each administration group during the initial 30 min. However, the intracellular concentration of these substrates generally decreased as the administration time exceeded 30 min (Figure 4A). For methotrexate, the intracellular concentration level was highest at 30 min, and then showed a certain decreasing trend with RPC, SPC, and BBRR administration (Figure 4B). The extracellular medium concentration of estrone-3-sulfate decreased gradually with the administration of RPC, SPC, BBR, and BBRR within 0–60 min of administration (Figure 4C). The concentration of rhodamine 123 in the extracellular medium decreased with the administration of SPC or BBRR when the administration time was 15–30 min, but it increased after 30 min (Figure 4D). The extracellular accumulation of topotecan reduced with BBRR when administered for 15–30 min, whereas it reduced with SPC during an administration period of 30–45 min (Figure 4E). The concentration of vinblastine in the extracellular medium of all dosing groups tended to increase with the duration of administration, while it decreased in each drug group when the administration time was 15–30 min (Figure 4F).

### 3.3 Protein transport assay

**FIGURE 5:**
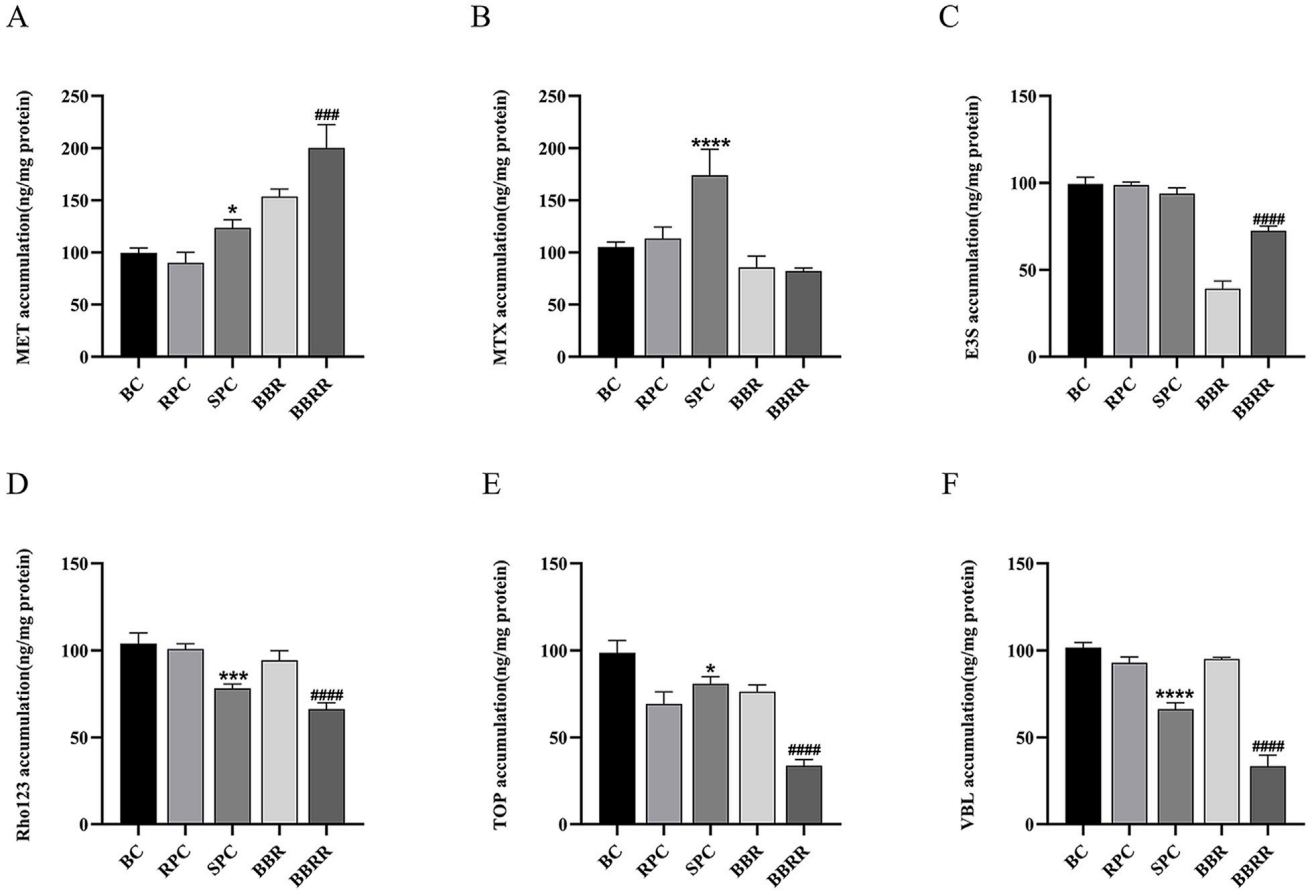
The effect of various solutions on each substrate accumulation transported by renal transport proteins. Blank control (BC), raw Phellodendri Chinensis cortex (RPC), Phellodendri Chinensis Cortex with salt-water processing (SPC), berberine (BBR), berberrubine (BBRR) **(A)** The effect of various solutions on the accumulation of MET transported by OCT2. **(B)** The effect of various solutions on the accumulation of MTX transported by OAT1. **(C)** The effect of various solutions on the accumulation of E3S transported by OAT3. **(D)** The effect of various solutions on the accumulation of Rho123 transported by P-gp. **(E)** The effect of various solutions on the accumulation of TOP transported by MATE1 and MATE2K. **(F)** The effect of various solutions on the accumulation of VBL transported by MRP2. Compared with the RPC group, ^*^ *p* < 0.05, ^**^ *p* < 0.01, ^***^ *p* < 0.001, ^****^ *p* < 0.0001; compared with the SPC group, ^#^ *p* < 0.05, ^##^ *p* < 0.01, ^###^ *p* < 0.001, ^####^ *p* < 0.0001 (n = 3).

The results showed a significant change in the effect of RPC, SPC, BBR, and BBRR on the trend of substrate concentration in cell lysates or extracellular media when the administration time was 30 min. Therefore, this point was selected to predict and analyze the effect of RPC, SPC, BBR, and BBRR on the transport capacity of transporter proteins OCT2, OAT1, OAT3, P-gp, MATE 1/2K, and MRP2 in the kidney. Figure 5 shows the effects of RPC, SPC, BBR, and BBRR on the transport capacity of renal transporter proteins by comparing the substrate accumulation. For OCT2, the MET accumulation significantly increased in SPC and BBRR groups compared to RPC and BBR groups (Figure 5A). For OAT1, the MTX accumulation increased in the SPC group compared to the RPC group (Figure 5B). For OAT3, the E3S accumulation decreased in the SPC group compared to the RPC group and significantly increased in the BBRR group compared to the BBR group (Figure 5C). For MATE1/2K, the TOP accumulation increased in the SPC group compared to the RPC group but decreased in the BBRR group compared to the BBR group (Figure 5E). For P-gp and MRP2, the accumulation of Rho123 and VBL decreased in SPC and BBRR groups compared to RPC and BBR groups (Figures 5D, F).

### 3.4 Effect of proteins expression

#### 3.4.1 Expression of OAT1, OAT3, OCT2 in HEK-293 cells

**FIGURE 6:**
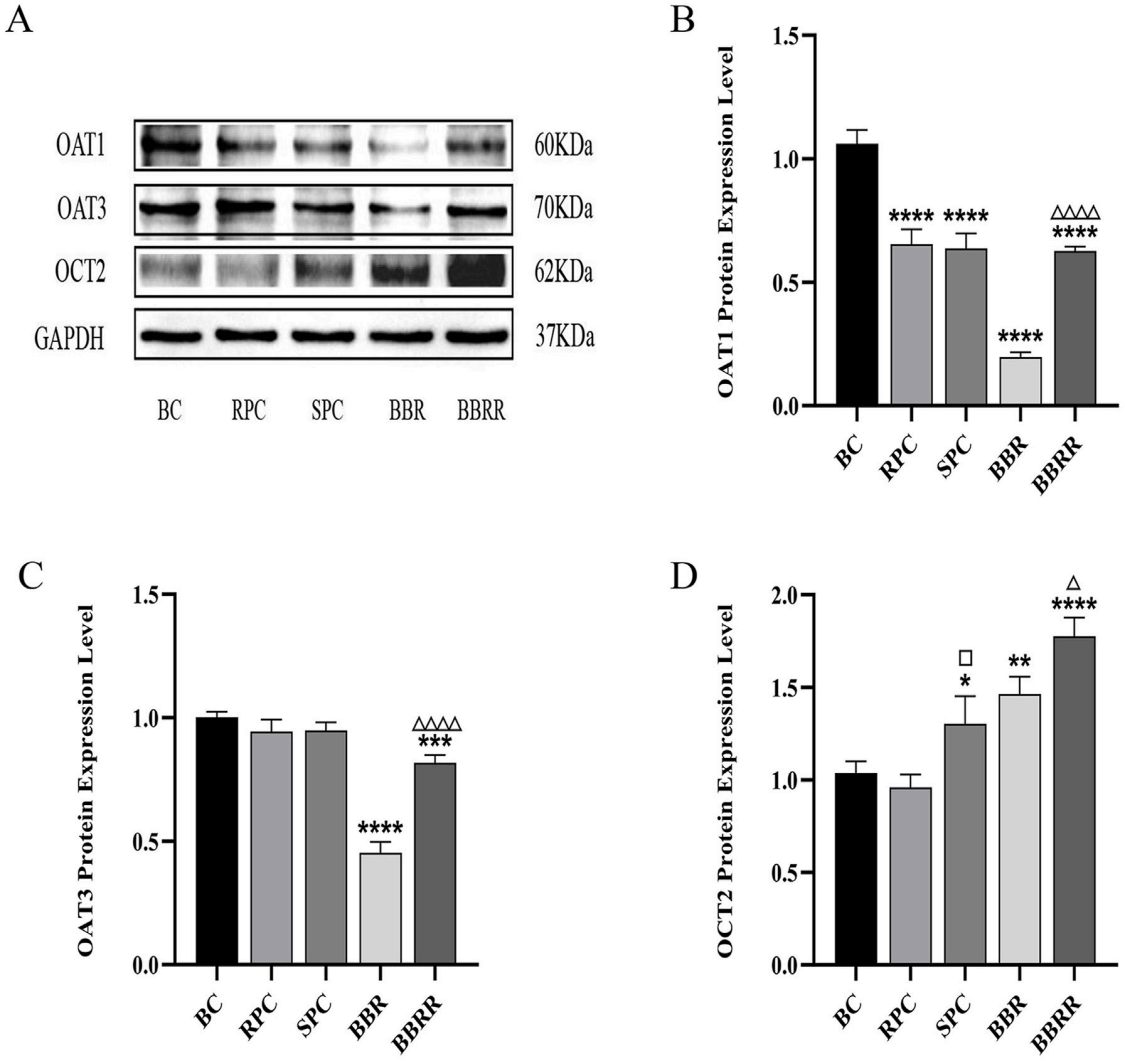
The effect of various solutions on the protein expression of OAT1, OAT3, OCT2. Blank control (BC), raw Phellodendri Chinensis cortex (RPC), Phellodendri Chinensis Cortex with salt-water processing (SPC), berberine (BBR), berberrubine (BBRR). **(A)** OAT1, OAT3, OCT2 protein expression bands in HEK-293 cells. **(B)** The effect of various solutions on OAT1 protein expression level. **(C)** The effect of various solutions on OAT3 protein expression level. **(D)** The effect of various solutions on OCT2 protein expression level. Compared with the BC group, ^*^ *p* < 0.05, ^**^ *p* < 0.01, ^***^ *p* < 0.001, ^****^ *p* < 0.0001; compared with the RPC group, ^□^ *p* < 0.05, ^□□^ *p* < 0.01, ^□□□^ *p* < 0.001, ^□□□□^ *p* < 0.0001; compared with the BBR group, ^△^ *p* < 0.05, ^△△^ *p* < 0.01, ^△△△^ *p* < 0.001, ^△△△△^ *p* < 0.0001.

Western blotting assay revealed a significant downregulation of OAT1 expression in the RPC, SPC, BBR, and BBRR groups compared to the blank control group. Similarly, the OAT3 expression markedly decreased in the BBR and BBRR groups, whereas no significant changes were observed in the expression of OAT1 and OAT3 in the SPC group compared to the RPC group. The expression of OAT1 and OAT3 increased significantly in the BBRR group compared to the BBR group. In contrast, the OCT2 expression significantly increased in the SPC, BBR, and BBRR groups compared to the blank control group. However, the SPC and BBRR groups exhibited a higher increase compared to the RPC group and the BBR group, respectively (Figure 6).

#### 3.4.2 Expression of MATE1, MATE2K, P-gp, MRP2 in HEK-293 cells

**FIGURE 7:**
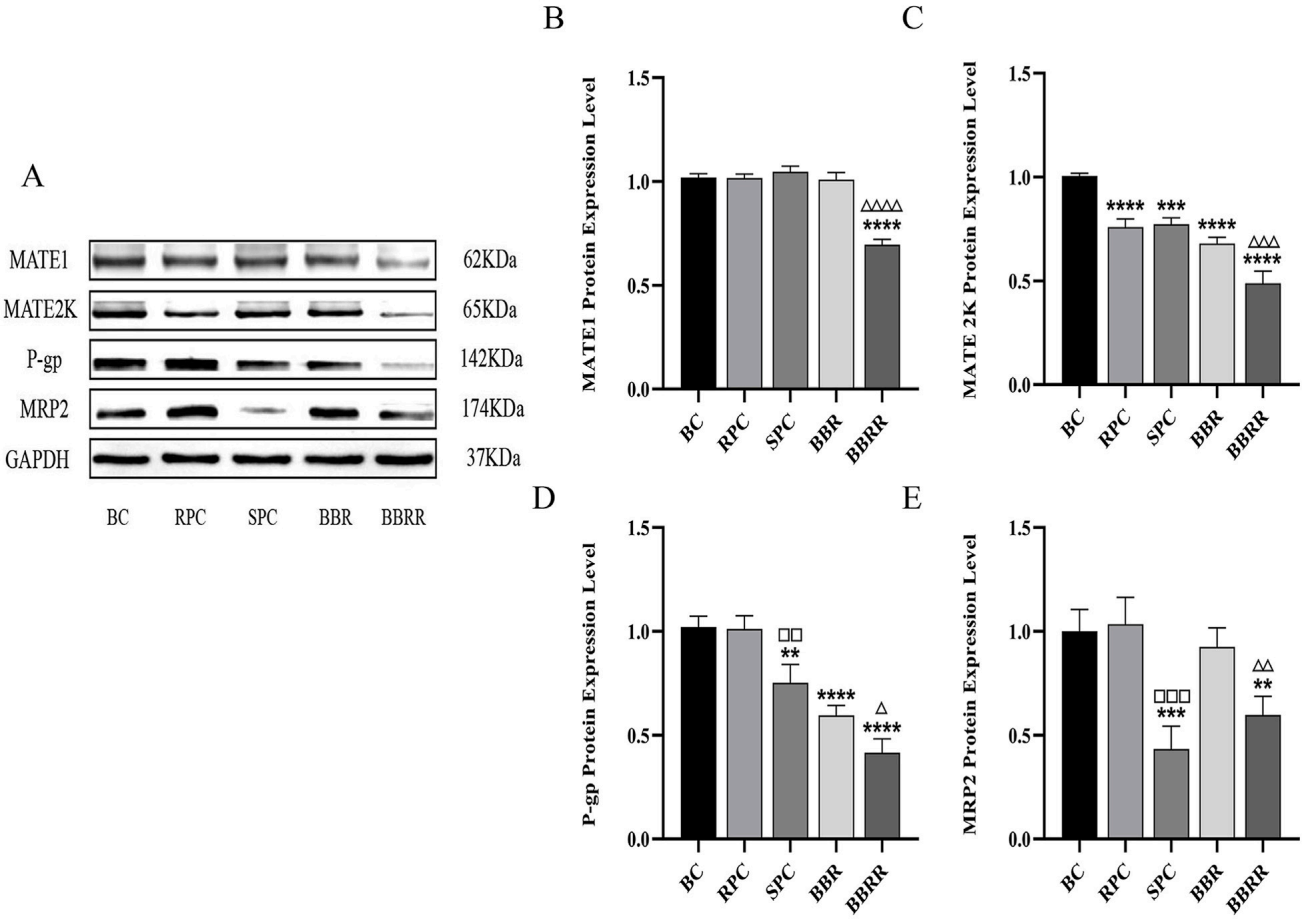
The effect of various solutions on the protein expression of MATE1, MATE2K, P-gp, MRP2. Blank control (BC), raw Phellodendri Chinensis cortex (RPC), Phellodendri Chinensis Cortex with salt-water processing (SPC), berberine (BBR), berberrubine (BBRR). **(A)** MATE1, MATE2K, P-gp, MRP2 protein expression bands in HEK-293 cells. **(B)** The effect of various solutions on MATE1 protein expression level. **(C)** The effect of various solutions on MATE2K protein expression level. **(D)** The effect of various solutions on P-gp protein expression level. **(E)** The effect of various solutions on MRP2 protein expression level. Compared with the BC group, ^*^ *p* < 0.05, ^**^ *p* < 0.01, ^***^ *p* < 0.001, ^****^ *p* < 0.0001; compared with the RPC group, ^□^ *p* < 0.05, ^□□^ *p* < 0.01, ^□□□^ *p* < 0.001, ^□□□□^ *p* < 0.0001; compared with the BBR group, ^△^ *p* < 0.05, ^△△^ *p* < 0.01, ^△△△^ *p* < 0.001, ^△△△△^ *p* < 0.0001.

Western blotting analysis indicated no significant difference between the RPC, SPC, and BBR groups and the blank control in the expression of MATE1. In contrast, the expression of MATE2K significantly decreased in the RPC, SPC, and BBR groups compared to the blank control. Additionally, a decline in the expression of both MATE1 and MATE2K was observed in the BBRR group compared to the BBR group. Simultaneously, the expression of P-gp was reduced in the SPC, BBR, and BBRR groups compared to the blank control, and the expression of MRP2 decreased in the SPC and BBRR groups compared to the blank control. Intra-group comparisons revealed that the expression of P-gp and MRP2 significantly reduced in the SPC group compared to the RPC group, and the BBRR group exhibited a similar decrease compared to the BBR group (Figure 7).

### 3.5 Effect of mRNA expression

#### 3.5.1 mRNA expression of OAT1, OAT3, OCT2

**FIGURE 8:**
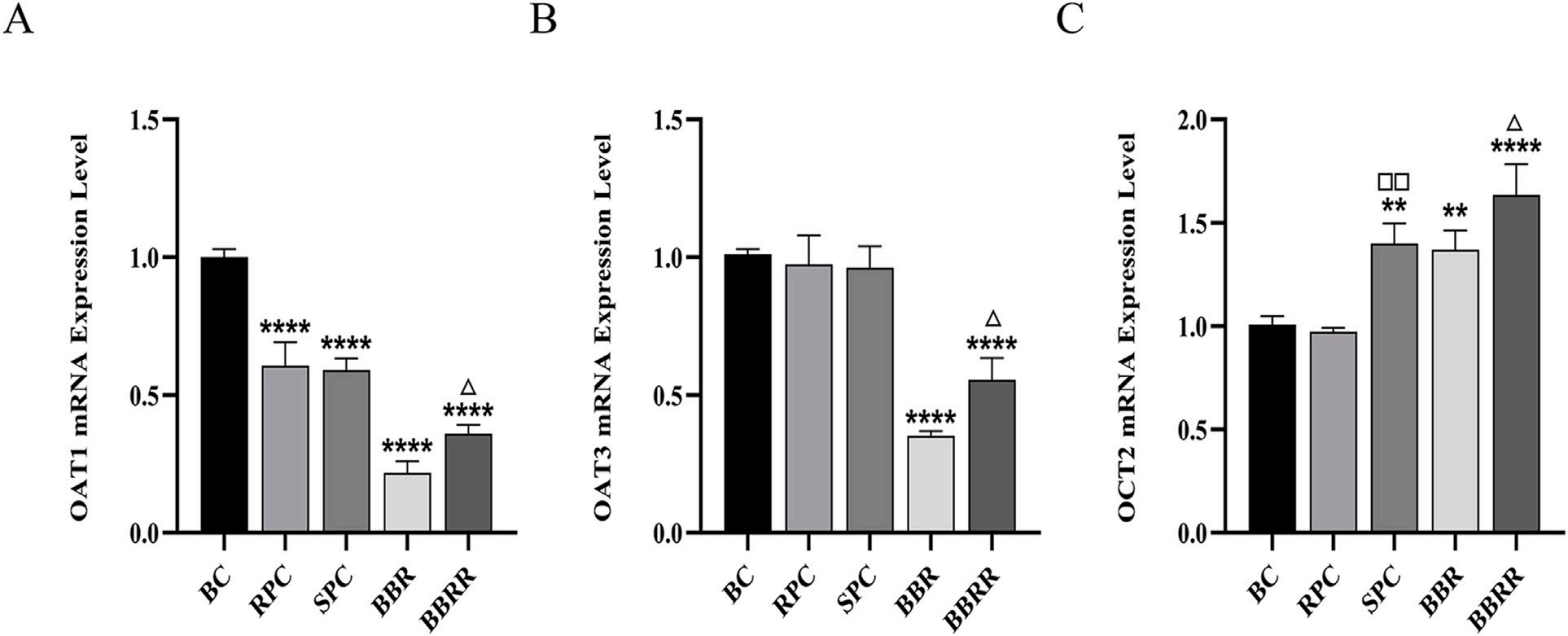
The effect of various solutions to OAT1, OAT3, OCT2 mRNA expression level. Blank control (BC), raw Phellodendri Chinensis cortex (RPC), Phellodendri Chinensis Cortex with salt-water processing (SPC), berberine (BBR), berberrubine (BBRR). **(A)** The effect of various solutions on OAT1 mRNA expression level. **(B)** The effect of various solutions on OAT3 mRNA expression level. **(C)** The effect of various solutions on OCT2 mRNA expression level Compared with the BC group, ^*^ *p* < 0.05, ^**^ *p* < 0.01, ^***^ *p* < 0.001, ^****^ *p* < 0.0001; compared with the RPC group, ^□^ *p* < 0.05, ^□□^ *p* < 0.01, ^□□□^ *p* < 0.001, ^□□□□^ *p* < 0.0001; compared with the BBR group, ^△^ *p* < 0.05, ^△△^ *p* < 0.01, ^△△△^ *p* < 0.001, ^△△△△^ *p* < 0.0001.

The RPC, SPC, BBR, and BBRR groups showed a significant decline in the expression of OAT1 mRNA in HEK-293 cells compared to the blank control group, and the BBRR group showed a decline in the expression of OAT1 and OAT3 mRNA compared to the BBR group. However, the expression of OCT2 mRNA increased in the SPC, BBR, and BBRR groups, with the SPC and BBRR groups exhibiting a significant increase compared to the RPC and BBR groups (Figure 8).

#### 3.5.2 mRNA expression of MATE1, MATE2K, P-gp, MRP2

**FIGURE 9:**
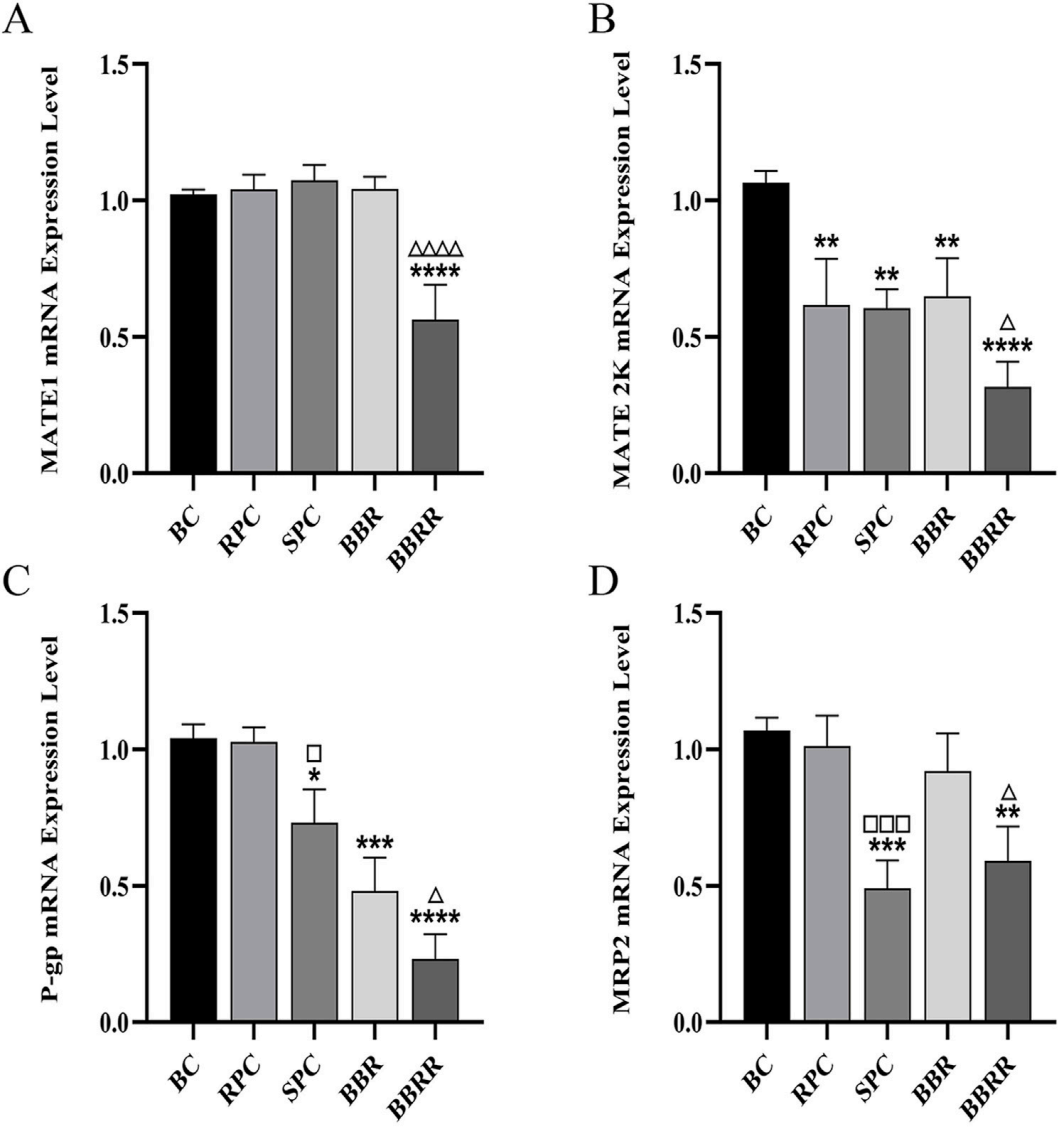
The effect of various solutions to MATE1, MATE2K, P-gp, MRP2 mRNA expression level. Blank control (BC), raw Phellodendri Chinensis cortex (RPC), Phellodendri Chinensis Cortex with salt-water processing (SPC), berberine (BBR), berberrubine (BBRR). **(A)** The effect of various solutions on MATE1 mRNA expression level. **(B)** The effect of various solutions on MATE2K mRNA expression level. **(C)** The effect of various solutions on P-gp mRNA expression level. **(D)** The effect of various solutions on MRP2 mRNA expression level Compared with the BC group, ^*^ *p* < 0.05, ^**^ *p* < 0.01, ^***^ *p* < 0.001, ^****^ *p* < 0.0001; compared with the RPC group, ^□^ *p* < 0.05, ^□□^ *p* < 0.01, ^□□□^ *p* < 0.001, ^□□□□^ *p* < 0.0001; compared with the BBR group, ^△^ *p* < 0.05, ^△△^ *p* < 0.01, ^△△△^ *p* < 0.001, ^△△△△^ *p* < 0.0001.

Compared to the blank control group, all of the RPC, SPC, BBR, and BBRR groups showed a significant decline in the expression of MATE2K, P-gp, and MRP2 mRNA in HEK-293 cells. The SPC and BBRR groups also showed a greater decline in the expression of P-gp and MRP2 mRNA compared to the RPC or BBRR groups (Figure 9).

## 4 Discussion

According to TCM theory, the term “kidney” refers not only to the physiological and anatomical organ but also to a characteristics whole that encompasses the kidney organ and its related functions. Therefore, our previous studies have already explored the therapeutic efficacy and regulatory functions of SPC on kidney-related diseases through using a variety of animal and cell models. In our pharmacological studies, we investigated the effects of different processed products of PC on the KYDP in rats. We found that SPC exhibited superior therapeutic effects compared to RPC, significantly enhancing antioxidant capacity and mitigating inflammatory responses in rats with KYDP (Ren et al., 2024). In relevant cell models, we also observed that the enrichment capacity of transformed alkaloids, such as BBRR in SPC, was markedly enhanced in the kidneys (Ge et al., 2024). These findings highlight the significant differences between SPC and other processed products or RPC, further illustrating the TCM processing theory of “entering into the kidney by processing with salt-water.” While the aforementioned studies have collectively demonstrated that SPC has a notable therapeutic effect on kidney diseases, the mechanisms by which the active metabolites in SPC enter the kidneys and exert their therapeutic effects remain complex and are not well-documented in literature. Therefore, we designed this experiment to preliminarily investigate the reasons for the enhanced renal targeting efficacy of SPC from the perspective of renal transporters in the physiological organ of the kidney.

The stir-frying temperature in the salt-water processing of PC was precisely maintained between 150°C and 160°C to cause notable changes in the structure and composition of the thermally unstable monomeric metabolite, BBR. This transformation resulted in the formation of another monomer effector, BBRR. BBR and BBRR are the primary alkaloidal sources of active metabolites in RPC and SPC, respectively. Consequently, the medicinal efficacy and pharmacokinetic properties of PC depend on its change before and after salt-water processing. For example, the distribution and bioavailability of PC’s active metabolites in rats were significantly altered following salt-water processing, which also influenced a variety of physiological and biochemical indices, such as energy and substance metabolism and the thyroid axis (Xu et al., 2015; Zhang et al., 2017; Lei et al., 2020). Moreover, SPC modulates the production of inflammatory factors such as CRH and ACTH in the blood and influences the expression of Bcl-2, Fas, and Caspase-3 in rat adrenal tissues, thereby effectively regulating the hypothalamic-pituitary-adrenal (HPA) axis and bolstering immune function (Jiang et al., 2024). These discrepancies in the effects of PC and SPC on the organism somewhat clarify the augmented kidney-targeting efficacy of SPC and the subsequent transformation of its clinical benefits. After the PC was processed with salt-water, the cation-carrying alkaloid effector BBR was partially converted to BBRR, which also functions as a cation-carrying alkaloid. The change may be partially related to the mechanism SPC to enhance renal targeting efficacy, induced by changes in the transporter activity and the expression of the relevant transporter proteins. However, the specific mechanisms by which SPC enhances renal targeting warrant further exploration. This study investigated the mechanisms through which SPC enhances renal targeting efficacy at the cellular and molecular levels. Additionally, SPC and BBRR influenced the expression and substrate uptake capacity of OCTS in HK-2 cells, as determined by ligand fishing and other experiments (Ge et al., 2024). This finding underscores the effect of chemical composition differences between RPC and SPC on the drugs’ ability to target renal transporter proteins, leading to divergent clinical efficacies and applications. This study hypothesized that the partial conversion of BBR to BBRR during salt-water processing may account for enhanced renal targeting efficacy. Therefore, this experiment established the BBR and BBRR administration groups to compare their effects on renal transporter proteins.

HEK-293 cells, derived from human embryonic kidney cells, express a diverse array of drug transporter proteins and metabolic enzymes, effectively emulating the physiological processes of drugs in the kidney (Henry et al., 2005). This characteristic renders them an optimal model for investigating drug transport mechanisms. Consequently, this study established an *in vitro* culture administration model utilizing HEK-293 cells to assess the effects of partial conversion of monomer active metabolites on renal transporter proteins with PC before and after the salt-water processing. In addition, HEK-293 cells are frequently used in studies related to carrier transport and have a relatively high transfection success rate. We will leverage the aforementioned advantages of HEK-293 cells to construct overexpressed protein models in subsequent experiments and further investigate the effects of PC on the relevant renal transporters before and after salt-water processing. The human body contains multiple families of transporter proteins, each with distinct members responsible for transporting various substances (Morrissey et al., 2013). Notably, OAT1, OAT3, OCT2, P-gp, MATE1, MATE2K, and MRP2 predominantly express in the kidney and play crucial roles in renal metabolism (Bendayan, 1996; Feng et al., 2010; Sun et al., 2014). OAT1, OAT3, and OCT2 play the role of transporter by mediating the entry of ionic metabolites with different charge attributes into the cell, thus affecting the renal reabsorption and excretion processes (Klaassen and Aleksunes, 2010; DeGroter et al., 2012). Driven by ATP hydrolysis, P-gp actively extrudes lipid-soluble cationic drugs from cells, diminishing their intracellular accumulation and consequently reducing renal damage inflicted by certain exogenous toxins (Leschziner et al., 2007). MATE1 and MATE2K mediate the excretion of organic cationic metabolites, exhibiting substantial overlaps in their substrate specificities (Masuda et al., 2006; Walsh et al., 2015). Despite differences in their renal enrichment, both transporters may participate in the excretion of substances. MRP2, with its broad substrate range, actively transports toxins or toxic drugs out of cells, offering renoprotection and detoxification. P-gp and MRP2 in the kidney can also interact with some alkaloid metabolites, such as berberine and aconitine, which in turn directly affects the metabolic process of drugs in the kidney and exogenous nephrotoxicity (Liu X. H. et al., 2013; Brodzicka et al., 2024). The modern research shows that human kidneys contain a variety of transporters, but their expression levels and enrichment degrees vary significantly. Some transporters, such as OAT2, OCT1 and OCT3, have low expression levels or are not expressed in the kidneys. Therefore, we have selected OAT1, OAT3, OCT2, P-gp, MATE1, MATE2K, and MRP2 to better elucidate the mechanism by which salt-water processing can enhance renal targeting efficacy. In addition, there are certain alkaloid metabolites, which may interact with renal proteins, affecting drug secretion and reabsorption, and thereby influencing drug bioavailability, therapeutic efficacy, and organ toxicity. After the PC was processed with salt-water, the cation-carrying alkaloid effector BBR was partially converted to BBRR, which also functions as a cation-carrying alkaloid. The change may be partially related to the mechanism SPC uses to enhance renal targeting efficacy, induced by changes in the transporter activity and the expression of the above-mentioned transporter proteins. Therefore, the above proteins were used in the subsequent experiments to investigate the effect of PC with salt-water processing on the absorption and excretion of drugs in the kidneys. We previously observed significant differences in the content of alkaloids taken up by HK-2 cells (Ge et al., 2023). This finding may be one of the reasons underlying the enhanced renal targeting of SPC. However, PC contains numerous metabolites in addition to alkaloids, which may also influence renal targeting efficacy. Therefore, we employed specific substrates of renal transporters as indicators for UPLC-TDQ-MS/MS analysis to gain a more comprehensive understanding. By comparing the differences in the content of substrates transported by each transporter, we explored the effects of SPC on the transport capacity of these transporters to further elucidate the potential mechanisms by which SPC enhances renal targeting efficacy.

Based on the selected representative transporter proteins in the kidney and related studies on the partial conversion of BBR, as the alkaloidal metabolite of PC, into BBRR before and after salt-water processing, molecular docking techniques were employed to predict the molecular-level interactions of BBR and BBRR with renal transporter proteins, including OAT1, OAT3, OCT2, MATE1, MATE2K, P-gp, and MRP2. This approach aimed to investigate the affinity of these two alkaloidal metabolites with the seven transporter proteins. Generally, when the binding energy between a receptor and ligand is lower than 0, the reaction is spontaneous. A lower binding energy indicates a better binding ability, with binding energies below −6 kcal/mol signifying strong binding activity, and those below −7 kcal/mol indicating even stronger binding activity (Meng et al., 2011; Fan et al., 2019; Lai et al., 2021), which also suggests higher potential interactions. From the perspective of BBR and BBRR docking with uptake transporter proteins, OCT2, as a cationic transporter protein, required less binding energy than OAT1 and OAT3 for docking with BBR and BBRR. Since the molecular docking results were consistent with the expectations, it was hypothesized that the alkaloidal metabolites in SPC could enhance the expression of OCT2. BBRR required significantly less binding energy than BBR did to dock with the anion-transporting proteins, *i*.*e*., OAT1 and OAT3. This suggested that BBRR may affect OAT1 and OAT3, in addition to OCT2. This effect may also be another mechanism by which SPC enhances renal targeting efficacy. Additionally, from the perspective of efflux transporter proteins, BBRR needed greater binding energies than BBR did to dock with P-gp, MATE1, MATE2K, and MRP2. Except for MATE2K, the binding of BBR and BBRR with MATE1, P-gp, and MRP2 was a strong spontaneous reaction, suggesting that the SPC’s renal targeting mechanism may be less relevant to the effect of BBR on MATE2K. Conversely, the effect of BBRR on MATE1, P-gp, and MRP2 may be another potential mechanism SPC uses to enhance renal-targeting effects. However, unlike OCT2, P-gp, and MRP2, the transport capability and protein expression levels of MATE1 and MATE2K, as determined from cellular transport, Western blotting, and RT-PCR assays between RPC and SPC groups, did not reveal a clear relationship between MATE1/2K and SPC in enhancing renal targeting efficacy. Additionally, molecular docking is a computer-based simulation predictive technique and cannot directly illustrate definitive results. Therefore, these two proteins were not selected for further investigation in the conclusion section. Nevertheless, we plan to construct hMATE1-HEK293 and hMATE2K-HEK293 cell models in future experiments to validate the relationship between the transporters MATE1/2K and the traditional theory of “entering into the kidney by processing with salt-water.”

This study also employed the UPLC-TDQ-MS/MS technique to determine trends in cellular uptake and efflux of substrates at different dosing times and to correlate the accumulation of the substrate transported by renal proteins of RPC, SPC, BBR, and BBRR administered. Therefore, it was hypothesized that SPC and BBRR cause more significant effects on the cation transporter protein OCT2. This may be due to OCT2’s higher affinity for BBRR, as the alkaloid metabolite of SPC; following the administration of SPC and BBRR, this greater affinity significantly increased OCT2’s binding to the substrate and improved its uptake capacity. This consequently led to an increased binding and transport of the substrate. Furthermore, this study predicted and analyzed the effect of RPC, SPC, BBR, and BBRR on the accumulation of substrates mediated by the transporter proteins OAT1 and OAT3. The results showed that none of the drug administration groups significantly affected the substrate transport capabilities of OAT1 and OAT3. Thus, it was hypothesized that the enhancement of renal targeting efficacy by SPC may not be significantly related to the effect of the BBR-to-BBRR conversion on the transport ability of anion transport proteins OAT1 and OAT3. Both SPC and BBRR significantly inhibited the activity of efflux transporter proteins, specifically P-gp and MRP2. This was evidenced by the decreased concentration of substrates in the extracellular culture medium and the decreased substrate accumulation mediated by these proteins after drug administration. This is because the partial conversion of BBR to BBRR could inhibit the ability of P-gp and MRP2 to transport the substrate, leading to prolonged drug retention in the kidneys. This process regulates the drug’s bioavailability in the organism and enhances its efficacy. Therefore, the study results suggest that the enhancement of renal targeting efficacy by PC after salt-water processing is related to the partial conversion of BBR to BBRR. This study investigated the effects of PC on the expression of renal transporter proteins and their mRNAs before and after salt-water processing using Western blotting and RT-PCR. The results further validated the speculations proposed in previous studies. Firstly, for the uptake transporter proteins, it was observed that both SPC and BBRR significantly affected the expression of OCT2 and its mRNA. However, SPC failed to significantly influence the expression of OAT1 and OAT3 and their mRNAs, when compared to RPC. Secondly, for the efflux transporter proteins, SPC significantly inhibited the expression of P-gp and MRP2 and their mRNAs more than RPC did. BBRR also exhibited significantly greater inhibitory effects than BBR on the expression of P-gp and MRP2 and their mRNAs. Therefore, it can be concluded that BBRR has a more pronounced inhibitory effect than BBR on the expression of efflux proteins, *i*.*e*., P-gp and MRP2, and their mRNAs in the kidneys, potentially leading to a greater ability to inhibit the efflux of certain exogenous drugs. The study results regarding the effects of SPC and BBRR on the expression of OCT2, P-gp, and MRP2 and their mRNAs are consistent with the findings of some previous studies on cell and protein transport. This suggests that the partial transformation of some active metabolites of PC after salt-water processing can affect the uptake and efflux of substrates mediated by OCT2, P-gp, and MRP2, and simultaneously influence the expression of their protein and mRNA. This indicates that the enhancing effect of PC after salt-water processing on renal targeting efficacy is related to its influence on the expression of certain renal transporter proteins. In conclusion, the enhancing effects of SPC on renal targeting efficacy can be attributed to drug-protein interactions between the uptake transporter protein (OCT2) and efflux transporter proteins (P-gp and MRP2) in the kidney and BBRR, which is the active alkaloid metabolite of SPC. When exploring the mechanisms of interactions between drugs and transporters, overexpressed protein models are often essential for elucidating the effects of drugs on transporters. However, in this experiment, our goal is to preliminarily screen the interactions between SPC and renal transporters at expression levels in the kidney. In future experiments, we plan to employ overexpressed protein models to further validate the mechanism by which SPC enhances renal targeting efficacy.

In addition, single active metabolites of TCMs, such as BBR and BBRR, can act as inhibitors or promoters of the expression of certain gene pathway proteins and their RNAs, thereby directly or indirectly affecting the expression of some uptake and efflux transporter proteins in the kidney (Ashrafizadeh et al., 2020; Sun et al., 2021). The enhancing effect of SPC on renal targeting efficacy is likely related to the effect of the inflations of other therapeutic metabolites in SPC on a variety of transporter proteins and their upstream and downstream pathways in the kidneys. However, few studies have addressed the interactive mechanism of SPC for expressing some uptake and efflux proteins and RNAs in the kidneys, as well as for their upstream and downstream gene pathways. This study elucidated the relationship between the enhancing effects of SPC on renal targeting efficacy and specific transporter proteins in the kidney. Additionally, it will construct HEK-293 cells with elevated expression of transporter proteins to validate the impact of SPC on particular substrates of these proteins, thereby further explaining the traditional processing theory that PC, when subjected to salt-water processing, can enhance the renal targeting efficacy of drugs.

## 5 Conclusion

This study demonstrated that SPC and BBRR significantly enhances the expression of the uptake transporter protein (OCT2), while inhibiting the expression of efflux proteins (P-gp and MRP2). This may explain why SPC has a more pronounced enhancing effect on renal targeting efficacy compared to RPC and BBR. The underlying mechanism involves the conversion of BBR to BBRR during the salt-water processing, which differentially affects these transporter proteins. Therefore, this study elucidated the cellular and protein-level mechanisms by which SPC enhances renal targeting efficacy and explained how salt-water processing of PC improves renal targeting efficacy, with a particular focus on the role of renal transporter proteins. These findings substantiate the traditional PCMM theory of “entering into the kidney by processing with salt-water.” Additionally, this study offers novel insights and methodologies for investigating the effects of PCMM on meridian tropism.

## Data Availability

The original contributions presented in the study are included in the article/supplementary material, further inquiries can be directed to the corresponding author.

## References

[B1] Ashrafizadeh M. Fekri H. S. Ahmadi Z. Farkhondeh T. Samarghandian S. (2020). Therapeutic and biological activities of berberine: the involvement of Nrf2 signaling pathway. J. Cell. Biochem. 121 (2), 1575–1585. 10.1002/jcb.29392 31609017

[B2] Bendayan R. (1996). Renal drug transport: a review. Pharmacotherapy 16 (6), 971–985. 10.1002/j.1875-9114.1996.tb03022x 8947968

[B3] Brodzicka A. Galanty A. Paśko P. (2024). Modulation of multidrug resistance transporters by food components and dietary supplements: implications for cancer therapy efficacy and safety. Curr. Issues. Mol. Biol. 46 (9), 9686–9706. 10.3390/CIMB46090576 39329928 PMC11430623

[B4] Bu X. W. Hao X. H. Zhang R. Y. Zhang M. J. Wang Z. (2024). Mechanism of qingrunfang-containing serum improving insulin resistance of HepG2 cells via branched-chain alpha-keto acid dehydrogenase regulation of branched-chain amino acids (BCAAs)/mTOR pathway. Chin. J. Exp. Tradit. Med. Formulae. 2 (1), 1–12. 10.13422/j.cnki.syfjx.20250201

[B5] Chen J. L. Song Y. P. Zhou Y. Z. Gong W. X. Qin X. M. (2024). Protective effect of Angelica sinensis water extract medicated serum on corticosterone-damaged PC12 cells through modulation of sphingolipid metabolic pathway. Drug Eval. Res. 47 (12), 2745–2756. 10.7501/j.issn.1674-6376.2024.12.005

[B6] Cheng E. M. Zhan Z. L. Zhang W. Yang H. J. Shen J. J. Peng H. S. (2019). Textual research of “Huangbo” in classical prescriptions. China J. Chin. Mat. Med. 44 (21), 4768–4771. 10.19540/j.cnki.cjcmm.2019.0909101 31872676

[B7] China Pharmacopoeia Commission (2020). Pharmacopeia of the people’s Republic of China. Beijing: China Medical Science Press, 318–319.

[B8] DeGroter M. K. Xia C. Q. Yang J. J. Kim R. B. (2012). Drug transporters in drug efficacy and toxicity. Annu. Rev. Pharmacol. Toxicol. 52, 249–273. 10.1146/annurev-pharmtox-010611-134529 21942630

[B9] Fan J. Fu A. Zhang L. (2019). Progress in molecular docking. Quant. Biol. 7, 83–89. 10.1007/s40484-019-0172-y

[B10] Fan S. M. Zhang C. L. Luo T. Wang J. Q. Tang Y. Yu L.Y. (2020). Study on Effect of Nourishing Yin and Clearing Heat of Zhimu-Huangbo Herb Pair Processed with Salt on Kidney-yin Deficiency Rats. Tradit. Chin. Drug. Res. Clin. Pharmacol. 31 (10), 1141–1146. 10.19378/j.issn.1003-9783.2020.10.002

[B11] Feng B. LaPerle J. L. Chang G. Varma M. (2010). Renal clearance in drug discovery and development: molecular descriptors, drug transporters and disease state. Expert Opin. Drug Metab. Toxicol. 6 (8), 939–952. 10.1517/17425255.2010.482930 20433402

[B12] Ge X. T. Zhao J. H. Ren W. J. Zhou Y. Zhang F. (2023). Difference of intake of alkaloids in renal tubular epithelial cells from different kinds of processed products of Phellodendri Chinensis Cortex. Chin. Tradit. Herb. Drugs 2023 54 (18), 5993–6000. 10.7501/j.issn.0253-2670.2023.18.017

[B13] Ge X. T. Zhao J. H. Ren W. J. Zhou Y. Chen Y. Jiang S. R. (2024). Alkaloid uptake pathways in renal tubular epithelial cells from different processed products of Phellodendri chinensis Cortex. J. Pharm. Biomed. Anal. 242, 116014. 10.1016/J.JPBA.2024.116014 38367517

[B14] Henry O. Perrier M. Kamen A. (2005). Metabolic flux analysis of HEK-293 cells in perfusion cultures for the production of adenoviral vectors. Metab. Eng. 7 (5), 467–476. 10.1016/j.ymben.2005.08.002 16198135

[B15] Huo X. K. Meng Q. Wang C. Y. Wu J. J. Zhu Y. N. Sun P. Y. (2020). Targeting renal OATs to develop renal protective agent from traditional Chinese medicines: protective effect of Apigenin against Imipenem‐induced nephrotoxicity. Phytother. Res. 34 (11), 2998–3010. 10.1002/ptr.6727 32468621

[B16] Jia T. Z. Zhang Z. L. Zhang X. L. Zhao R. H. Li F. (2013). Science of Chinese meteria Medica processing. second edition. Shanghai: Shanghai Scientific and Technical Publishers, 196–198.

[B17] Jia Y. Liu Z. H. Wang C. Y. Meng Q. Huo X. K. Liu Q. (2016). P-gp, MRP2 and OAT1/OAT3 mediate the drug-drug interaction between resveratrol and methotrexate. Toxicol. Appl. Pharmacol. 306 (6), 27–35. 10.1016/j.taap.2016.06.030 27377006

[B18] Jiang S. R. Li L. Zhao J. H. Ge X. T. Ren W. J. Zhou Y. (2024). Study on the regulation of hypothalamic-pituitary-adrenal Axis (HPA Axis) in rats with kidney-yin deficiency syndrome by the raw and salt-water processing of Phellodendri chinensis cortex. Int. J. Pharmacol. 20 (3), 455–471. 10.3923/ijp.2024.455.471

[B19] Klaassen C. D. Aleksunes L. M. (2010). Xenobiotic, bile acid, and cholesterol transporters: function and regulation. Pharmacol. Rev. 62 (1), 1–96. 10.1124/pr.109.002014 20103563 PMC2835398

[B20] Lai G. H. Wang F. Zhou F. Xiang T. T. Wen L. Wu Z.J. (2021). Mechanism of Gusuibu (Drynariae Rhizoma)‐Buguzhi (Psoraleae Fructus) Drug Pair on Treatment of Bone Metastasis Cancer Pain Based on Network Pharmacology and Molecular Docking. J. Hunan Univ. Chin. Med. 41 (09), 1372–1380. 10.3969/j.issn.1674-070X.2021.09.011

[B21] Lei X. F. Shan G. S. Zhang F. Liu P. P. Meng L. Jia T. Z. (2020). Determination and comparison of alkaloids and triterpenes among tissues after oral administration of crude and processed Phellodendri Chinensis Cortex by UPLC-QqQ-MS. Nat. Prod. Res. 34 (9), 1337–1340. 10.1080/14786419.2018.1560293 30663377

[B22] Leschziner G. D. Andrew T. Pirmohamed M. Johnson M. R. (2007). ABCB1 genotype and PGP expression, function and therapeutic drug response: a critical review and recommendations for future research. Pharmacogenomics J. 7 (3), 154–179. 10.1038/sj.tpj.6500413 16969364

[B23] Li L. Zhang C. Zheng W. Yu Y. Q. Zhang F. Gao H. (2021). UPLC-QqQ-MS-based quantitative analysis on changes of ten components between the raw and salt-water processed Phellodendron chinense Cortex. Chin. Tradit. Pat. Med. 43 (11), 3082–3088. 10.3969/j.issn.1001-1528.2021.11.026

[B24] Liu P. P. Zhang F. Zhao Y. Xu S. Sun N. Jia T. Z. (2013a). Advances in the study salt-water processing of tradition Chinese medicine. China Pharm. 24 (43), 4101–4104. 10.6039/j.issn.1001-0408.2013.43.24

[B25] Liu X. H. Yu N. Di Q. Li L. C. Zhang X. H. (2013b). Effect of berberine on the expression of P-glycoprotein in the epileptic rat’s brain tissue. J. Clin. Neurol. 26 (03), 191–194.

[B26] Masuda S. Terada T. Yonezawa A. Tanihara Y. Kishimoto K. Katsura T. (2006). Identification and functional characterization of a new human kidney–specific H^+^/organic cation antiporter, kidney-specific multidrug and toxin extrusion 2. J. Am. Soc. Nephrol. 17 (8), 2127–2135. 10.1681/ASN.2006030205 16807400

[B27] Meng X. Y. Zhang H. X. Mezei M. Cui M. (2011). Molecular docking: a powerful approach for structure-based drug discovery. Curr. Comput. Aided. Drug. Des. 7 (2), 146–157. 10.2174/157340911795677602 21534921 PMC3151162

[B28] Morrissey K. M. Stocker S. L. Wittwer M. B. Xu L. Giacomini K. M. (2013). Renal transporters in drug development. Annu. Rev. Pharmacol. Toxicol. 53 (1), 503–529. 10.1146/annurev-pharmtox-011112-140317 23140242

[B29] Morrissey K. M. Wen C. C. Johns S. J. Zhang L. Huang S. M. Giacomini K. M. (2012). The UCSF‐fda transportal: a public drug transporter database. Clin. Pharmacol. Ther. 92 (5), 545–546. 10.1038/clpt.2012.44 23085876 PMC3974775

[B30] Nies T. A. Hofmann U. Resch C. Elke S. Maria R. Schwab M. (2017). Proton pump inhibitors inhibit metformin uptake by organic cation transporters (OCTs). PLoS ONE 6 (7), 22163. 10.1371/journal.pone.0022163 PMC3136501 21779389

[B31] Pohl A. Lage H. Müller P. Pomorski T. Herrmann A. (2002). Transport of phosphatidylserine via MDR1 (multidrug resistance 1) P-glycoprotein in a human gastric carcinoma cell line. Biochem. J. 365 (1), 259–268. 10.1042/BJ20011880 12071854 PMC1222671

[B32] Qi D. L. Jia T. Z. Lian L. (2010). Change of components in Phellodendri chinensis cortex after processing. Chin. Tradit. Pat. Med. 32 (3), 443–447.

[B33] Que H. Y. Luo Q. L. Wang N. Zhang X. Gu J. Gong P. Y. (2022). Research progress on processing historical evolution and mechanism of salt-water processed Phellodendri Chinensis Cortex and predictive analysis on its quality marker(Q-Marker). Chin. Tradit. Pat. Med. 53 (22), 7242–7253. 10.7501/j.issn.0253-2670.2022.22.029

[B34] Ren W. J. Zhao J. H. Li L. Ge X. T. Zhou Y. Chen Y. (2024). Energy metabolism, immune function, and intestinal flora in rats with kidney-yin deficiency treated with raw or saltwater-processed Phellodendron chinense Schneid. J. Tradit. Chin. Med. Sci. 11 (04), 488–499. 10.1016/j.jtcms.2024.09.008

[B35] Sun C. Qi J. F. Zhang N. Yu W. H. Wang Y. H. (2014). Research advances in roles of membrane transporters in renal drug disposition. Chin. J. Pharmacol. Toxicol. 28 (04), 625–631. 10.3867/j.issn.1000-3002.2014.04.024

[B36] Sun R. Kong B. Yang N. Cao B. Feng D. Yu X. (2021). The hypoglycemic effect of berberine and berberrubine involves modulation of intestinal farnesoid X receptor signaling pathway and inhibition of hepatic gluconeogenesis. Drug. Metab. Dispos. 49 (3), 276–286. 10.1124/DMD.120.000215 33376148

[B37] Van Aubel R. A. Koenderink J. B. Peters J. G. Van C. H. Russel F. G. (1999). Mechanisms and interaction of vinblastine and reduced glutathione transport in membrane vesicles by the rabbit multidrug resistance protein Mrp2 expressed in insect cells. Mol. Pharmacol. 56 (4), 714–719. 10.1016/s0026-895x(24)12532-1 10496953

[B38] Walsh D. R. Nolin T. D. Friedman P. A. (2015). Drug transporters and Na+/H+ exchange regulatory factor PSD-95/drosophila discs large/ZO-1 proteins. Pharmacol. Rev. 67 (3), 656–680. 10.1124/pr.115.010728 26092975 PMC4485015

[B39] Wittwer M. B. Zur A. A. Khuri N. Kido Y. Kosaka A. Zhang X. X. (2013). Discovery of potent, selective multidrug and toxin extrusion transporter 1 (MATE1, SLC47A1) inhibitors through prescription drug profiling and computational modeling. J. Med. Chem. 56 (3), 781–795. 10.1021/jm301302s 23241029 PMC4068829

[B40] Xiao G. Q. Rowbottom C. Boiselle C. Gan L. S. (2018). Fampridine is a substrate and inhibitor of human OCT2, but not of human MATE1, or MATE2K. Pharm. Res. 35 (2), 159–168. 10.1007/s11095-018-2445-y 29915999

[B41] Xu S. Zhang F. Liu P. P. Jia T. Z. (2015). Effects of processing on Phellodendri chinensis cortex based on material and energy metabolism. J. Chin. Med. Mat. 38 (09), 1835–1841. 10.13863/j.issn1001-4454.2015.09.011 26930977

[B42] Yang L. Q. Wu Y. M. Li W. (2024). Inhibition of HMGB1-induced inflammation in BEAS-2B cells by Thirty-six swings of kan Clam San. Chin. Tradit. Pat. Med. 46 (12), 4151–4156. 10.3969/j.issn.1001-1528.2024.12.041

[B43] Yang N. Sun R. B. Zhao Y. Q. He J. Zhen L. Guo J. (2016). High fat diet aggravates the nephrotoxicity of berberrubine by influencing on its pharmacokinetic profile. Environ. Toxicol. Pharmacol. 46 (5), 319–327. 10.1016/j.etap.2016.08.003 27525563

[B44] Zhang F. Gao H. Xu G. Zhao Y. Jia T. Z. (2013). Study on change of alkaloids from Phellodendri chinensis cortex while processing. Chin. Med. Pharm. 3 (1), 43–45.

[B45] Zhang F. Li L. Zhao J. H. Ge X. T. Gao H. Jia T. Z. (2022). The effects of salt-water processing of Phellodendri chinensis cortex on the enhancement of kidney absorption of the main alkaloids. Nat. Prod. Commun. 17 (2), 1–10. 10.1177/1934578X221076218

[B46] Zhang F. Xu S. Liu P. P. Jia T.Z. (2017). Effects of Different Processed Products of Phellodendron chinense Cortex on Thyroid Gland and Adrenocortical Function of Kidney‐Yin Deficiency Model Rats with Hyperthyroidism. China Pharm. 28 (1), 27–30. 10.6039/j.issn.1001-0408.2017.01.07

[B47] Zhang J. Liu K. X. (2010). Intestinal absorption and renal excretion mediated by transporters and the relationship with drug-drug interaction. Acta Pharm. Sin. 45 (9), 1089–1094. 10.16438/j.0513-4870.2010.09.011 21351563

[B48] Zhao S. X. Xia Q. M. (2022). Advances in Research on the Pharmacological Effects of Bupleurum. Asia-Pacific Tradit. Med. 18 (3), 228–232. 10.11954/ytctyy,202203052

[B49] Zhu F. Q. Wu F. J. Ma Y. Liu G. J. Li Z. Sun Y. (2011). Decrease in the production of beta-amyloid by berberine inhibition of the expression of beta-secretase in HEK293 cells. BMC Neurosci. 12 (2), 125–128. 10.1186/1471-2202-12-125 22152059 PMC3253691

